# Genitourinary and Syphilitic Diseases1This “copy” was prepared by Dr. Daggett but a short time before his death, is nearly all pen written, and was, in all probability, his last literary work.—Editor.

**Published:** 1904-02

**Authors:** Byron H. Daggett

**Affiliations:** Buffalo, N. Y.


### PROGRESS IN MEDICAL SCIENCE.

### Genitourinary and Syphilitic Diseases.[1]

CONDUCTED BY BYRON H. DAGGETT, M. D., Buffalo, N. Y.

### THE TECHNIQUE OF PROSTATECTOMY.

NICHOLAS SENN (*American Jour. of Dermatology and Genito-urinary Diseases,* September, 1903,) states that the most feasible route by which to attack the diseased prostate has not been definitely settled. The suprapubic method has been deemed the easiest, most efficient and safest, but a large experience and more extended trial has apparently decided in favor of the perineal route. The perineal operation, from the anatomical standpoint, is most rational and will probably stand the test of time and experience.

1. This "copy" was prepared by Dr. Daggett but a short time before his death, is nearly all pen written, and was, in all probability, his last literary work.—EDITOR.

Removal of the enlarged prostate does not always relieve urinary obstruction and infection; it is often necessary to establish free drainage, after the removal of the prostate, in order to successfully combat co-existing complications. All prostates can not be removed by enucleation, and morcellement or piecemeal removal is necessary.

It is the rule of surgery to operate as little as possible in the dark in important anatomical localities. Large prostates in obese subjects are very difficult to reach and to illumine through the perineum, hence there is a great advantage in an incision that will expose the prostate in the freest possible manner to sight and touch and this is best done by combining the median with two lateral incisions. The median incision lays bare the membranous portion of the urethra and the lateral incisions are carried from the lower pole of the incision to a point half way between the anal margin and the tuberosity of the ischium, cutting through the same structures involved in the lateral operation for stone in the bladder. The wound is deepened by blunt instruments and hemorrhage arrested as it occurs. In this way the rectum is detached until the membranous portion of the urethra and the lower segment of the prostate can be seen and felt in the apex of the wound. The wound is dilated and the rectum drawn back by using narrow, flat and deep retractors, thus exposing the urethra and dependent portion of the prostate. The membranous portion of the urethra is opened on a grooved staff, the left index finger passed into the bladder and used as a blunt hook with which to draw the bladder downward and forward, and incise the capsule of the gland transversely; introduce the left index finger into the incision and peal out the left prostatic lobe; reverse the hand and peel out the right lobe. Sometimes this is done easily, other times it is difficult, and at other times it is impossible; avoid violence and recklessness. Enucleation may be facilitated by traction of the gland with forceps. If feeling is impracticable morcellement must be resorted to. The finger in the bladder serves as a valuable retractor and guide. An assistant holds and manipulates the traction forceps while the operator does the cutting with blunt-pointed scissors, well curved on the flat. Enough tissue should be removed to relieve obstruction.

Complete cystotomy should be done for irrigation in complicated cases and is preferable to tearing into the urethra by attempting to do prostatectomy without a perineal cystotomy. Bladder drainage should be continued until the urine becomes normal.

FALLACIES OF CYSTOSCOPY.

SCHMIDT, L. E., (*Chicago Therapeutic Gazette,* November, 1902,) says that overrating and not understanding the defects often discredit a valuable method of diagnosis or treatment. The cystoscope may deceive the observer and lead to wrong conclusions and even cause unwarranted operations.

Morbid conditions are, in a great majority of bladder cases, located about the trigonum and the internal urethral orifice. These parts are often covered by a protruding fold of the urethral mucosa giving the field of view a reddish tinge, which if unrecognised may convey the idea that an inflammation exists here. In order to determine the question the beak of the cystoscope is placed in different positions and the views compared. The position of the ureteral openings varies quite often in distance from the urethral orifice or they may be covered by the orificial folds, and for these reasons they may be overlooked.

Inflammation or edema roughens the surface of the trigonum and obscures the ureteral openings and poking efforts to find them may do damage. If there is doubt as to the locality of the ureteral openings, introduce the finger into the vagina or rectum and press the trigone towards the cystoscopic window and if the orifice is not found watch for the swirl or spurting out of the urine. Methylene blue, given by the mouth, will give the urine a green color by which the ureteral output can be distinguished from the fluid distending the bladder. The presence of pus in the urine renders the examination more difficult. The urethral orifice is very close to the cystoscopic window, which magnifies, and in this way changes the actual appearance to the eye of the observer. To give a normal size and appearance, with the Netze cystoscope, the object should be about 3 cm. from the prism.

The cystoscope shank is rigid, straight and large, which changes the formation and appearance of the orificial tissue. Inspection of the normal urethral orifice through a suprapubic opening affirms this statement.. The edematous folds of the internal urethral orifice, magnified through the optical apparatus misleads the observer. These folds appear often in great numbers, they are not transparent and appear to be quite rigid. The polyp, located at the orifice is transparent, very movable, floating in water, and a loop of bloodvessels running through it can be seen.

Preliminary to a Bottini operation the cystoscope is used to decide whether a barrier exists which can be severed with a galvano-cautery blade with probable success. It is of paramount importance that this barrier, bar, or lobe should spring from the bladder and not from the prostatic urethra. Nodules and lax membrane from the prostatic urethra may be pushed through and over the urethral orifice by the straight tube, and this mass mistaken for a cystic abnormality.

If this error is made galvano-cautery is dangerous and useless, as the prostatic mass draws back into the prostatic urethra, so that the cuts are made in the prostatic urethra and often lead to serious consequences.

It may be demonstrated that the barrier belongs to the bladder by its covering of bladder mucosa, indicating its presence in the bladder before the introduction of the cystoscope; the urethral mass is covered with urethral mucosa. The *bas-fond* behind the barrier must be deep. If the ureteral orifices cannot be seen there is a grave suspicion that the tumor was pushed into the bladder.

In nervous subjects the touch of the instrument causes circumscribed, energetic contraction, which may be mistaken for a trabeculated bladder. In a trabeculated bladder the trabeculation is uniform over the fundus and vertex, and the protrusion of muscular bundles is constant.

Edema bullosum may be diagnosticated as a malignant tumor. Tumors covered with incrustrations may be taken for calculi, and calculi imbedded in the mucosa for tumors. Pedunculated tumors, often flattened, may be taken for malignant growths on account of their apparent broad base. Movement of these tumors will reveal their true condition.

Varicosities are sometimes seen in the lateral walls of the bladder and should be recognised. Cystoscopic burns are sometimes taken for idiopathic ulcers. Their location, absence of inflammatory action and quick healing indicate their nature.

The direct view without enlargement by optical apparatus is apt to lead to misconception because it covers a very small area.

---

### GALVANO-CAUSTIC TREATMENT OF PROSTATIC HYPERTROPHY AFTER BOTTINI.

VOGEL, (*Jour. des Practicines,* p. 361, 1903,) ; (*Am. Med.,* Sept. 5, 1903,) reports several cases operated upon with the galvano-cautery, with full and empty bladders. The first, a man 71 years old, died half an hour after operation without any apparent cause. An examination showed the incision very superficial, supposedly due to the cooling of the knife by the borated fluid in the bladder. Second patient operated upon, first with full bladder, without improvement; second time with empty bladder. Death followed in forty-eight hours from peritonitis, caused by a cut through a fold in the bladder. Vogel recommends operating on a full bladder, distending with air or fluid.

---

### INDICATIONS AND CONTRAINDICATIONS FOR PROSTATECTOMY.

BAZET, L., (*American Med., September,* 1903,) states that reaction is taking place against prostatectomy, due to indiscriminate operation. In selected cases the operation is indicated, and should be done through a perineal opening. The size and character of the prostate should be determined by combined rectal and abdominal examination, by cystoscopy and the use of the biconder sound. The symptoms of only dysuria and nocturnal frequency, with enlarged prostate, do not call for immediate prostatectomy. Acute detention demands prompt relief by prostatectomy, in the presence of enlarged prostate; if small, defer operation. In chronic, incomplete retention operate, if the prostate is large. The larger ratio of failures result from operation in this condition. Contraindications are great age, debility, infection and nephritis.

---

### VARICOCELE.

VARICOCELE, (Editorial *American Med.,* September, 1903,) rarely causes serious atrophy of the testicle or serious impairment of the sexual function. There are three established operative procedures; subcutaneous ligation of the veins; ablation of the dependent portion of the scrotum to shorten the support of the testicle, and open incision. The last is the one usually employed.

The United States army surgeons, according to Thornburgh, (*Boston Medical and Surgical Journal,* August 27, 1903,) describe the suprapubic method of operation as follows: briefly the operation consists in making a 3 cm. incision extending through the skin and deep fascia, whose center is over the external ring of the affected side, parallel to Poupart's ligament. The cord is exposed, the vas dissected free to near the testicle, the latter together with the enlarged and tortuous veins, is brought up into the wound by gentle traction. The veins are ligated in two places with No. 3 cumolised catgut, the intervening segment of veins resected, and the cut ends approximated as in the open scrotal incision. The deep structures and skin wound are approximated with No. 1 cumolised catgut. Thornburgh had slight suppuration in one instance and this he attributed to the use of chromic catgut which was not readily absorbed and caused irritation. In army practice the recruit is operated upon when the varicocele is as large or larger than the testicle of the sound side, otherwise operation is not deemed necessary. The advantages claimed for the suprapubic site are more certainty of disinfection, lessened liability to stitch abscess and other infection, more favorable site for the application and retention of an aseptic dressing, and lessened probability of sinus formation.

---

THE OCULAR MANIFESTATIONS IN CHRONIC BRIGHT'S DISEASE.

De Schweinitz, (*Med.*, Dec., 1902,) mentions the ocular lesions which may be associated with, or caused by various forms of Bright's disease: (1) complete blindness without ophthalmoscopic lesions, or at least without the presence of lesions more or less suggestive of disease of the kidneys, generally called uremic amaurosis, and most often seen in acute nephritis, but also in acute exacerbations of chronic renal disease; (2) various types of retinitis and neuroretinitis, to which the descriptive term "albuminuric" is commonly applied, and which are most often seen in association with chronic forms of kidney disease; (3) alterations in the caliber and relation to the retinal vessels owing to sclerotic changes in their walls, with or without hemorrhages and exudates in the retina, seen in association with those forms of renal disease in which vascular changes are evident elsewhere in the body; also isolated hemorrhages and exudates, without marked vessel-wall changes; (4) alterations in the uveal tract, particularly in the choroid and iris; (5) some varieties of cataract; (6) paresis and paralysis of the ocular muscles, particularly the superior oblique and the external rectus; (7) recurring subconjunctival hemorrhages.—*International Medical Magazine*, March, 1903.

---

VARIATIONS IN ALBUMIN, URIC ACID, AND TOTAL ACIDITY IN URINES WITH PERMANENT OR INCONSTANT ALBUMINURIA.

Daremberg and Moriez (*Revue de Med.*, No. 9, 1902; *Gaz. des Hop.*, 1902, p. 1,098,) have studied the quantitative variations in certain elements found in the urine and the changes which occur in the total acidity under these conditions. They arrived at the following conclusions:

All albuminurias which occur in patients in fairly good condition either entirely disappear or are at a minimum in the morning. In these different forms the time of maximal elimination of albumin is extremely variable. In the case of permanent albuminuria there is always some albumin found in the urine between noon and 5 p. m. In those who have a morning disappearance the albumin shows itself between 10 a. m. and 10 p. m.; but most frequently between noon and 6 p. m. In inconstant albuminuria there is no particular periodicity in the appearance of the albumin in the cases observed. In that with matutinal absence some are influenced by the digestion, while others are not; of these the first demand alkalies, the second arsenic. Massage is equally unavailing in both varieties. In the inconstant form the amounts of uric acid, the total acidity, and quantity of albumin are occasionally proportional; sometimes inversely proportional. The differences between the maximum and minimum of uric acid are much greater in albuminuric patients with a morning disappearance than in healthy persons. In healthy individuals the hourly curve of elimination of uric acid and total acidity are concordant. This is not the case in those albuminurics who show the morning absence of albumin. The hours of maximal and minimal elimination of uric acid and degree of acidity are constant in well persons; but very inconstant in those with albuminuric. Patients troubled with albuminuria of the inconstant type may increase in weight, even during a period of augmented amounts of albumin.—*International Medical Magazine,* March, 1903.

---

THE IMPORTANCE OF HYALIN CASTS IN THE DIAGNOSIS AND PROGNOSIS OF RENAL DISEASES.

DEFENDORF, A. R., (*Yale Med. Jour.,* December, 1902,) says that purely hyalin casts have been found in large percentages in individuals who are apparently healthy and without other evidence of kidney disorder. These percentages increase with the advance of age. This, in the absence of *post-mortem* observations, has led to a more or less general belief that hyalin casts in urine of individuals past 50 are without significance. A series of 48 autopsies in which urine examinations had been made some time previous to death, show that casts were present in 41 cases and chronic nephritis in 46 cases, permitting the inference that hyalin casts in these cases were distinctly an indication of an organic disease of the kidneys. The 5 disease conditions in which hyalin casts exist alone or with finely granular casts are: the atrophic stage of chronic parenchymatous nephritis, chronic interstitial nephritis, chronic diffuse nephritis, senile interstitial nephritis, and chronic passive congestion of the kidneys. The prognosis of these conditions, with the exception of passive congestion, depends as much upon the general clinical condition of the patient, especially in reference to cardiac and vascular disease, as upon the character of the kidney lesion.—*International Medical Magazine,* March, 1903.

---

THE authorities at Butler, Pa., issued an order December 7, 1903, which will greatly lessen the danger of typhoid fever, and which will to a great extent protect the towns in the valleys below Butler.

